# Supplementary material for: Metasurface‐Enabled Active‐Like Passive Radar
Source: Adv Sci (Weinh). 2026 May 12:e75629. Online ahead of print. doi: 10.1002/advs.75629 (PMC13335912; doi:10.1002/advs.75629)
Supplement: Supplementary file 1 — Supporting File 1: advs75629‐sup‐0001‐SuppMat.docx. [file ADVS-9999-e75629-s007.docx]

**Supplementary Information for**

**Metasurface-Enabled Active-Like Passive Radar**

Mingyi Li^1+^, Jiawen Xu^1+^, Hanting Zhao^1+^, Xu Zhao^1^, Yanjin Chen^1^, Tie Jun Cui^2, 3^, Vincenzo Galdi^4^, and Lianlin Li^1, 5^

^1^ State Key Laboratory of Photonics and Communications, Peking University, Beijing 100871, China

^2^ State Key Laboratory of Millimeter Waves, Southeast University, Nanjing 210096, China

^3^ Suzhou Laboratory, Suzhou 215004, China

^4^ Fields & Waves Lab, Department of Engineering, University of Sannio, I-82100 Benevento, Italy

^5^ Pazhou Laboratory (Huangpu), Guangzhou, Guangdong 510555, China

**Outline:**

**Supplementary Note 1.** Design and Implementation of the One-Bit STC-PM

**Supplementary Note 2.** Derivation of the K-Component Spatiotemporal Modal Decomposition

**Supplementary Note 3.** Design of Spatial Modes and Derivation of the Signal Model

**Supplementary Note 4.** Derivation of the Interference Suppression Mechanism

**Supplementary Note 5.** Analysis of MEPR Detection Performance Under Varying Conditions

**Supplementary Note 6.** UAV Platform Used in Experiments

**Supplementary Note 7.** Greedy Search Algorithm for Rapid UAV Detection and Tracking

**Supplementary Note 8.** Link-budget Analysis for MEPR

**Supplementary Note 1. Design and Implementation of the One-Bit STC-PM**

The STC-PM serves as the core component of the MEPR platform, enabling active control of EM waves through dynamic spatiotemporal modulation of ambient wireless signals. By continuously tuning both the reflection amplitude and phase distribution in both space and time, the STC-PM transforms a conventional passive radar aperture into an active and reconfigurable sensing platform. In principle, an ideal STC-PM should provide independent tunability of reflection amplitude and phase within the range of $[0, 1] \times[0^{\circ}, 180^{\circ}]$. For practical feasibility and operational stability, a one-bit configuration was implemented to achieve fast and reliable modulation. Each meta-atom supports two reflection states with nearly identical amplitude and opposite phase, thereby realizing binary phase control. The metasurface operates at 5.48 GHz, a frequency commonly used in wireless communication and radar sensing.

As shown in Supplementary Fig. 1a, the fabricated metasurface panel consists of a $32 \times24$ array of programmable meta-atoms organized into twelve $8 \times8$ subpanels, forming an effective aperture of $0.781 \times0.586 m^{2}$. The front view shows the uniform meta-atom array, whereas the back view highlights the integrated control hardware, including the FPGA and MCU modules, and the detailed view further shows the MCU board, which employs eight shift registers (SN74LV595APW). Supplementary Fig. 1b presents a photograph of a metasurface subpanel sample, together with the three-dimensional schematic and the layered structure of a meta-atom. Each meta-atom has a lateral size of 24.4 mm × 24.4 mm and consists of a five-layer stack. **Layer 1: Cu** mainly comprises a 17 mm × 17 mm square metallic patch. **Layer 2: F4BM,** with a thickness of 1.2 mm, is an F4BM dielectric substrate with a relative permittivity of 2.55 and a loss tangent of 0.0019. A 90° bent metallic phase-shifting line located near the edge of the metallic patch is connected to **Layer 3: Cu** (the metallic ground plane) through a metallized via. A commercial PIN diode is mounted across the gap between the phase-shifting line and the metallic patch. The PIN diode used in this design is SMP1345-079LF from Skyworks. Beneath the metallic ground plane, an additional FR-4 dielectric substrate with a thickness of 0.4 mm is introduced for bias-circuit routing, corresponding to **Layer 4: FR-4**. One end of the bias line is connected to the metallic patch, while the other end is connected to the external voltage control line on **Layer 5: Cu** through a metallized via passing through the substrate stack. It should be noted that this metallized via is not electrically connected to the metallic ground plane. In addition, to ensure effective isolation between the RF signal and the DC bias signal, an inductor with L = 33 nH is inserted between the metallic patch and the metallized via.

The EM response of the meta-atoms was characterized both numerically and experimentally, as illustrated in Supplementary Fig. 1c. Full-wave simulations were conducted using CST Studio Suite 2017 (https://www.3ds.com/it/products/simulia/cst-studio-suite), and the results were validated through free-space measurements of a representative metasurface subpanel. The ON and OFF states of the diode correspond to two distinct resonant conditions, producing a phase contrast of approximately 180° at 5.48 GHz with minimal amplitude variation. The measured reflection characteristics closely match the simulated results, confirming that the binary phase modulation scheme provides stable and reliable performance across the operational band. This one-bit configuration thus forms the physical foundation of the spatiotemporal coding functionality used in the MEPR platform.

**

**

**Supplementary Fig. 1 | Structure and system architecture of the one-bit STC-PM. a,** Photographs of the fabricated metasurface panel. The front view shows the uniform meta-atom array, and the back view reveals the integrated control hardware, including the FPGA and MCU modules; the right panel further shows a detailed view of the MCU board, which employs eight shift registers (SN74LV595APW). **b,** Sample of a metasurface subpanel and the meta-atom design. The left image shows a metasurface subpanel sample; the middle panel illustrates the three-dimensional structure of a meta-atom; the right panel shows its five-layer configuration, including the metallic patch, PIN diode, and inductor. **c,** Simulated and measured reflection characteristics of the meta-atom in the ON and OFF states, showing an approximately 180° phase difference at 5.48 GHz with minimal amplitude variation. **d,** Schematic of the complete control and measurement architecture for the STC-PM.

The complete control and measurement architecture is illustrated in Supplementary Fig. 1d. Each metasurface subpanel contains eight 8-bit shift registers (SN74LV595APW; see Supplementary Fig. 1a for the hardware implementation), where each register sequentially drives eight PIN diodes. The entire array of 768 diodes is partitioned into twelve 8 × 8 subpanels, each controlled by an individual microcontroller unit (MCU), for a total of 12 MCUs, and driven in real time by FPGA-based biasing circuits. The host computer transmits the preconfigured spatiotemporal coding sequences and timing schedules to the FPGA via Ethernet. The Universal Software Radio Peripheral (USRP) communicates with the host computer through a high-speed optical fiber link to ensure high-bandwidth data transmission. The USRP serves as the hardware-level timing reference, generating microsecond-scale GPIO trigger pulses to command the FPGA to sequentially switch coding states according to the preconfigured spatiotemporal sequence and to synchronize signal acquisition. The entire coding-switching and data-acquisition process is synchronized by the USRP at a sub-microsecond level, ensuring precise temporal alignment and stable real-time spatiotemporal modulation during radar operation.

During each STC switching and data acquisition process, the system operates as follows. The host computer first preloads a one-bit bitstream of dimensions 32 × 24 × 1000 into the FPGA, corresponding to 1000 space–time coding states. The USRP then issues a GPIO trigger to initiate the STC sequence. Under FPGA control, the metasurface performs 1000 coding transitions at a rate of 2.5 µs per code, while the USRP simultaneously acquires backscattered signals from two receiving channels ($R_{1}$ and $R_{2}$) at a 10 MHz sampling rate, yielding 25,000 samples per acquisition window. The tight synchronization between modulation and signal acquisition maintains phase coherence at the microsecond scale, enabling precise far-field beam scanning and accurate wireless signal reception. This design ensures deterministic timing and stable operation throughout the entire spatiotemporal modulation process, thereby demonstrating the STC-PM as a robust and reconfigurable platform for active EM sensing.

**Supplementary Note 2. Derivation of the K-Component Spatiotemporal Modal Decomposition**

To establish the physical basis of Equation (1) in the main text, we start from the fundamental EM response of an STC-PM and derive the system response $y(t;\boldsymbol{r},\boldsymbol{r}_{s})$.

We consider an STC-PM composed of $N$ independently controllable meta-atoms, each exhibiting a time-varying EM response $\Gamma\left( n,t \right)$, where $n$ is the spatial index. The incident signal $s\left( t;\boldsymbol{r}_{s} \right)$ originates from a wireless source at position $\boldsymbol{r}_{s}$, with frequency spectrum $\tilde{s}\left( \omega\right)$. The transfer function describing the propagation from the source to the $n$-th meta-atom and then to the observation point $\boldsymbol{r}$ is denoted as $\tilde{H}_{\boldsymbol{r}_{s}\to n\to\boldsymbol{r}}(\omega)$. The total field observed at $\boldsymbol{r}$ can therefore be written as

| $y(t;\boldsymbol{r},\boldsymbol{r}_{s}).=s(t;\boldsymbol{r}_{s})\Gamma(n,t)= [\sum_{n=1}^{N} \int d\omega\text{ }\tilde{s}(\omega)\tilde{H}_{\boldsymbol{r}_{s}\to n\to\boldsymbol{r}}(\omega)e^{j\omega t}]\Gamma(n,t).$ | (S1) |
| --- | --- |

To describe the internal modulation structure of the metasurface, we introduce a *K*-component spatiotemporal modal decomposition, in which each meta-atom response is represented as a finite sum of separable spatial and temporal basis functions:

| $\Gamma(n,t)=\sum_{k=1}^{K} f_{k}(n)c_{k}(t),$ | (S2) |
| --- | --- |

where $f_{k}\left( n \right)$ and $c_{k}\left( t \right)$ denote the $k$-th spatial and temporal modal functions, respectively, and $K$ is the total number of spatiotemporal modes.

Substituting Equation (S2) into Equation (S1), yields

| $\begin{matrix} y\left( t;\boldsymbol{r},\boldsymbol{r}_{s} \right) & =\left[ \sum_{n=1}^{N} \int d\omega\text{ }\tilde{s}\left( \omega\right)\tilde{H}_{\boldsymbol{r}_{s}\to n\to\boldsymbol{r}}\left( \omega\right)e^{j\omega t} \right]\left[ \sum_{k=1}^{K} f_{k}\left( n \right)c_{k}\left( t \right) \right]. \\ & \end{matrix}$ | (S3) |
| --- | --- |

By regrouping terms, Equation (S3) can be equivalently rewritten as

| $y(t;\boldsymbol{r},\boldsymbol{r}_{s})=\sum_{k=1}^{K} [\int d\omega\text{ }e^{j\omega t}\tilde{s}(\omega)\sum_{n=1}^{N} \tilde{H}_{\boldsymbol{r}_{s}\to n\to\boldsymbol{r}}(\omega)f_{k}(n)]c_{k}(t).$ | (S4) |
| --- | --- |

We now define the effective modal transfer function

| $\tilde{H}_{k}(\omega;\boldsymbol{r},\boldsymbol{r}_{s})=\sum_{n=1}^{N} \tilde{H}_{\boldsymbol{r}_{s}\to n\to\boldsymbol{r}}(\omega)f_{k}(n),$ | (S5) |
| --- | --- |

whose time-domain counterpart is

| $f_{k}(t;\boldsymbol{r},\boldsymbol{r}_{s})=\mathcal{F}^{-1}\text{ }[\tilde{H}_{k}(\omega;\boldsymbol{r},\boldsymbol{r}_{s})],$ | (S6) |
| --- | --- |

with $\mathcal{F}^{-1}$ denoting the inverse Fourier transform.

Using the convolution theorem, the system response becomes

| $y(t;\boldsymbol{r},\boldsymbol{r}_{s})=\sum_{k=1}^{K} c_{k}(t)\text{ }[f_{k}(t;\boldsymbol{r},\boldsymbol{r}_{s})\otimes s(t;\boldsymbol{r}_{s})].$ | (S7) |
| --- | --- |

When the temporal reference is shifted from the metasurface plane to the observation point, a propagation delay $\tau_{m\to\boldsymbol{r}}$ is introduced, leading to $y(t;\boldsymbol{r},\boldsymbol{r}_{s})=\sum_{k=1}^{K} c_{k}\left( t-\tau_{m\to\boldsymbol{r}} \right)\left[ f_{k}\left( t-\tau_{m\to\boldsymbol{r}};\boldsymbol{r} \right)\otimes s(t-\tau_{m\to\boldsymbol{r}}) \right].$

Equation (S7) corresponds directly to Equation (1) in the main text. It shows that the STC-PM acts as a superposition of $K$ independent spatiotemporal modulation channels, each characterized by a temporal coding function $c_{k}\left( t \right)$ and a spatial impulse response $f_{k}\left( t;\boldsymbol{r},\boldsymbol{r}_{s} \right).$ This formulation establishes an explicit link between the element-wise behavior $\Gamma\left( n,t \right)$ and the macroscopic system response.

If the temporal modes are chosen as complex exponentials,

| $c_{k}(t)=e^{j\omega_{k}t}, k=1,2,\ldots,K,$ | (S8) |
| --- | --- |

Equation (S2) reduces to a Fourier-type expansion,

| $\Gamma(n,t)=\sum_{k=1}^{K} f_{k}(n)e^{j\omega_{k}t},$ | (S9) |
| --- | --- |

where each mode corresponds to a harmonic component at frequency $\omega_{k}$. Substituting Equation (S8) into Equation (S3) yields

| $y(t;\boldsymbol{r},\boldsymbol{r}_{s})=\sum_{k=1}^{K} [\int d\omega\text{ }e^{j\omega t}\tilde{s}(\omega)\sum_{n=1}^{N} \tilde{H}_{\boldsymbol{r}_{s}\to n\to\boldsymbol{r}}(\omega)f_{k}(n)]e^{j\omega_{k}t}.$ | (S10) |
| --- | --- |

Equation (S10) reveals that each frequency component $\omega_{k}$ corresponds to an independent spatiotemporal modulation channel, with spatial weighting defined by $f_{k}\left( n \right)$. If the spatial modes are designed to form mutually independent radiation beams, different frequency components are radiated along distinct spatial directions, naturally realizing a frequency-scanning STC-PM antenna.

Therefore, Equation (S7) provides a general theoretical framework that unifies the description of spatiotemporal modulation and connects the metasurface coding dynamics with observable macroscopic behaviors such as beam steering and frequency-scanning operation.

**Supplementary Note 3. Design of Spatial Modes and Derivation of the Signal Model**

This Note presents the theoretical and design framework for the spatial modes used in the MEPR approach. We first establish the principle of spatial beam focusing, then derive practical dual-beam spatial modes for STC-PMs, and finally link these modes to the received signals to obtain the system-level model used in the main text.

***Principle of Spatial Beam Focusing***

$\tilde{H}_{\boldsymbol{r}_{s}\to n\to\boldsymbol{r}}(\omega)$denotes the frequency-domain channel response of the $k$-th spatial mode in the absence of metasurface modulation, representing the propagation of a signal from the source at $r_{s}$, through the $n$-th meta-atom, to the observation point $\boldsymbol{r}$. Using a Green’s function formulation, the channel response along this path can be expressed as

| $\tilde{H}_{\boldsymbol{r}_{s}\to n\to\boldsymbol{r}}(\omega)=G(\boldsymbol{r}_{n},\boldsymbol{r}_{s};\omega)\text{ }G(\boldsymbol{r},\boldsymbol{r}_{n};\omega),$ | (S11) |
| --- | --- |

where $G\left( \boldsymbol{r},\boldsymbol{r}^{'} \right)=e^{-jk_{0}\mid\boldsymbol{r}-\boldsymbol{r}^{'}\mid}/\left( 4\pi\left| \boldsymbol{r}-\boldsymbol{r}^{'} \right| \right)$is the free-space Green’s function, with $k_{0}=\omega/c$ being the wavenumber and $c$ the speed of light. The overall frequency-domain channel response of the $k$-th spatial mode can then be obtained by superposing the contributions from all $N$elements:

| $\tilde{F}_{k}(\omega;\boldsymbol{r},\boldsymbol{r}_{s})=\sum_{n=1}^{N} f_{k}(n)\tilde{H}_{\boldsymbol{r}_{s}\to n\to\boldsymbol{r}}(\omega),$ | (S12) |
| --- | --- |

where $f_{k}(n)$ defines the spatial mode distribution of the $n$-th element under mode $k$. By properly designing $f_{k}(n)$, constructive interference can be achieved at the desired location, enabling spatial beam focusing.

***Design of Dual-Beam Spatial Modes***

Two spatial modes, $f_{1}$ and $f_{2}$, are used to establish independent channels. Mode $f_{1}$ forms a stable link between a designated source and the reference receiver $R_{1}$. Mode $f_{2}$ produces a probing beam steered or focused toward the region of interest for target sensing.

For convenience we define normalized mode weights

| $f_{1}^{'}\left( n \right)=\frac{f_{1}(n)}{\underset{n}{max}\mid f_{1}(n)\mid}, f_{2}^{'}(n)=\frac{f_{2}(n)}{\underset{n}{max}\mid f_{2}(n)\mid}.$ | (S13) |
| --- | --- |

**(1) Design of** $\boldsymbol{f}_{\mathbf{1}}^{\mathbf{'}}\left( \boldsymbol{n} \right)$ **for Focusing at** $\boldsymbol{R}_{\boldsymbol{1}}$

To achieve constructive interference at $\boldsymbol{r}_{1}$, the phase of $f_{1}^{'}\left( n \right)$ is set to cancel the propagation phase from the source to the element and from the element to $R_{1}$:

| $\angle f_{1}^{'}(n)=-k_{0}(\mid\boldsymbol{r}_{n}-\boldsymbol{r}_{s}\mid+\mid\boldsymbol{r}_{1}-\boldsymbol{r}_{n}\mid)+\phi_{0}, n=1,2,\ldots,N,$ | (S14) |
| --- | --- |

with $\phi_{0}$a reference phase, taken as zero without loss of generality. No apodization is applied to this link, hence

| $\mid f_{1}^{'}(n)\mid=1, n=1,2,\ldots,N$. | (S15) |
| --- | --- |

**(2) Design of** $\boldsymbol{f}_{\mathbf{2}}^{\mathbf{'}}\mathbf{(}\boldsymbol{n}\mathbf{)}$ **for Near-Field Focusing**

For focusing at a near-field point ***r***, the phase is similarly chosen as

| $\angle f_{2}^{'}(n)=-k_{0}(\mid\boldsymbol{r}_{n}-\boldsymbol{r}_{s}\mid+\mid\boldsymbol{r}-\boldsymbol{r}_{n}\mid)+\phi_{0}, n=1,2,\ldots,N.$ | (S16) |
| --- | --- |

To suppress sidelobes, we apply a separable two-dimensional Hamming apodization. Index $n$ maps to $\left( p_{n},q_{n} \right)$ on a $P \times Q$ grid. The amplitude profile is

| $\mid f_{2}^{'}(n)\mid=w_{P}(p_{n})\text{ }w_{Q}(q_{n})\text{ }$ | (S17) |
| --- | --- |

With

| $\left\{ \begin{aligned} w_{P}(p)=0.54-0.46cos(\frac{2\pi(p-1)}{P-1}), \\ w_{Q}(q)=0.54-0.46cos(\frac{2\pi(q-1)}{Q-1}). \end{aligned} \right.$ | (S18) |
| --- | --- |

This taper lowers sidelobe levels while maintaining a narrow main lobe.

**(3) Design of** $\boldsymbol{f}_{\mathbf{2}}^{\mathbf{'}}\mathbf{(}\boldsymbol{n}\mathbf{)}$ **for Far-Field Focusing**

In the far-field, for $\mid\boldsymbol{r}\mid\gg D^{2}/\lambda$with aperture $D,$ the distance is approximated by

| $\mid\boldsymbol{r}-\boldsymbol{r}_{n}\mid\approx\mid\boldsymbol{r}\mid-\hat{\boldsymbol{r}}\cdot\boldsymbol{r}_{n},$ | (S19) |
| --- | --- |

where $\hat{\boldsymbol{r}}$is the unit vector toward $\boldsymbol{r}$. The phase reduces to a linear gradient

| $\angle f_{2}^{'}(n)\approx-k_{0}(\mid\mathbf{r}_{n}-\mathbf{r}_{s}\mid-\hat{\mathbf{r}}\cdot\mathbf{r}_{n})+\phi_{0}, n=1,2,\ldots,N,$ | (S20) |
| --- | --- |

with $\phi_{0}=0$. The amplitude $|f_{2}^{'}(n)$| follows the same Hamming taper as in Equation (S17).

***Determination of the STC Sequence***

We now determine $\Gamma\left( n,t \right)$ given the target spatial modes. Let $\max_{n} \left| f_{k}\left( n \right) \right|$ denote the peak magnitude of mode k.

**(1) Ideal STC**

For an ideal STC device, amplitude and phase are continuously tunable in $\left[ 0,1 \right]$ and $\left[ 0^{\circ},{180}^{\circ} \right]$. Taking the magnitude of Equation (S2) yields

| $\sum_{k=1}^{K} {(\underset{n}{max}\mid f_{k}(n)\mid)}^{2}\leq1.$ | (S21) |
| --- | --- |

For $K=2$, let $a=\max_{n} \left| f_{1}\left( n \right) \right|$ and $b=\max_{n} \left| f_{2}\left( n \right) \right|$. A common choice is $a^{2}+b^{2}=1$. The space–time sequence is then calculated by

| $\Gamma(n,t)=\sum_{k=1}^{K} f_{k}(n)\text{ }c_{k}(t),$ | (S22) |
| --- | --- |

with $c_{k}(t)$ the temporal codes.

**(2) One-bit STC**

A one-bit device enforces unit magnitude and binary phase $\{0^{\circ},{180}^{\circ}\}$, so $\Gamma\left( n,t \right)\in\{+1,-1\}$. Since $\Gamma\left( n,t \right)$ is real while the $c_{k}\left( t \right)$ are complex and independent, conjugate terms appear, which tightens the bound to

| $\sum_{k=1}^{K} {(\underset{n}{max}\mid f_{k}(n)\mid)}^{2}\leq0.5.$ | (S23) |
| --- | --- |

For $K=2$ we empirically use $a^{2}+b^{2}=0.4$. Given the desired complex $f_{1}\left( n \right)$ and $f_{2}\left( n \right)$, the binary sequence $\Gamma\left( n,t \right)$ is obtained by

| $\underset{\Gamma(n,t)\in\{+1,-1\}}{min}(\mid f_{1}(n)-\int\Gamma(n,t)\text{ }c_{1}^{*}(t)\text{ }dt\mid^{2}+\mid f_{2}(n)-\int\Gamma(n,t)\text{ }c_{2}^{*}(t)\text{ }dt\mid^{2}),$ | (S24) |
| --- | --- |

which we solve efficiently using a binary genetic algorithm. This produces a one-bit sequence that best approximates the ideal multimode excitation under hardware constraints.

***Signal Model***

Under a narrowband assumption, the $k$-th spatial mode $\tilde{F}_{k}(\omega;\boldsymbol{r},\boldsymbol{r}_{s})$ in Equation (S12) can be treated as a complex constant equal to the modal radiation gain $\mathrm{at}$ $\boldsymbol{r}$:

| $\tilde{F}_{k}(\omega;\boldsymbol{r},\boldsymbol{r}_{s})=g_{k}=\sum_{n=1}^{N} f_{k}(n)\text{ }\tilde{H}_{\boldsymbol{r}_{s}\to n\to\boldsymbol{r}}(\omega).$ | (S25) |
| --- | --- |

Including the propagation delay $\tau_{m\to\boldsymbol{r}}$, the impulse response is approximated as

| $f_{k}(t;\boldsymbol{r},\boldsymbol{r}_{s})\approx g_{k}\text{ }\delta(t-\tau_{m\to\boldsymbol{r}}).$ | (S26) |
| --- | --- |

Substituting Equation (S26) into Equation (S7) yields

| $y(t;\boldsymbol{r},\boldsymbol{r}_{s})=\sum_{k=1}^{K} c_{k}(t)\text{ }[f_{k}(t;\boldsymbol{r},\boldsymbol{r}_{s})\otimes s(t;\boldsymbol{r}_{s})]\approx\sum_{k=1}^{K} G_{k}\text{ }c_{k}(t-\tau_{m\to\boldsymbol{r}})\text{ }s(t-\tau_{m\to\boldsymbol{r}};\boldsymbol{r}_{s}),$ | (S27) |
| --- | --- |

where $c_{k}\left( t \right)$ are the $k$-th temporal codes and $s(t;\boldsymbol{r}_{s})$ is the source waveform.

For the two-receiver architecture, mode 1 focuses the reference link at $R_{1}$ and mode 2 illuminates the target at $\boldsymbol{r}_{u}$. Receiver $R_{2}$ is directional and oriented toward the target, so it does not respond to direct radiation from the STC-PM. The received signals reduce to

| $y_{1}(t)\approx G_{1}\text{ }c_{1}(t-\tau_{m\to\boldsymbol{r}_{1}})\text{ }s(t-\tau_{m\to\boldsymbol{r}_{1}})+n_{1}(t),$ | (S28) |
| --- | --- |
| $y_{2}(t)\approx\alpha_{u}\text{ }G_{2}\text{ }c_{2}(t-\tau_{m\to\boldsymbol{r}_{u}\to\boldsymbol{r}_{2}})\text{ }s(t-\tau_{m\to\boldsymbol{r}_{u}\to\boldsymbol{r}_{2}})+n_{2}(t),$ | (S29) |

where $\tau_{m\to\boldsymbol{r}_{1}}$ and $\tau_{m\to\mathbf{r}_{u}\to\boldsymbol{r}_{2}}$ are the relevant delays, $\alpha_{u}$ is the target reflection response, $G_{1,2}$ are the modal gains, and $n_{1,2}(t)$ collect all STC-PM-irrelevant contributions.

**Supplementary Note 4. Derivation of the Interference Suppression Mechanism**


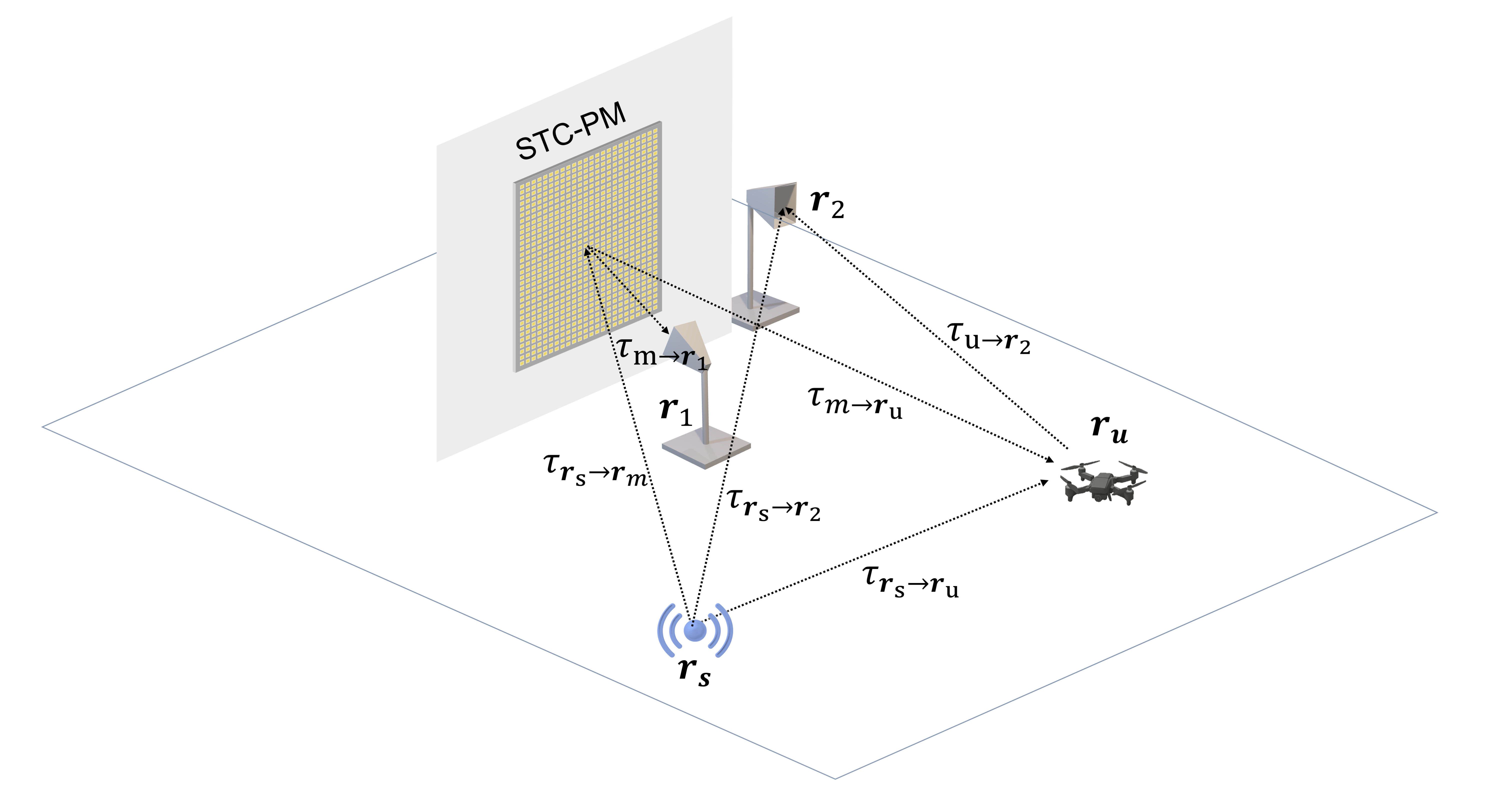


**Supplementary Figure 2** | Schematic illustration of delay relationships in the MEPR signal model.

Supplementary Table 1 summarizes the definitions and physical meanings of all symbols used in this Note, including received signals, radiation gains, temporal modes, propagation delays, and noise terms that appear in the MEPR signal model and in the interference cancellation procedure. To aid interpretation, Supplementary Fig. 2 provides a schematic of the delay relationships in the system, highlighting the paths associated with the metasurface-reference link, the metasurface-target-receiver link, and the direct-path terms. We define the time instant $t=0$as the moment when the STC begins its space–time coding transition.

### Supplementary Table 1. Main notation utilized throughout this supplementary note

| Symbol | Description |
| --- | --- |
| $y_{1}(t),y_{2}(t)$ | Received signals at receiver $R_{1}$ and receiver $R_{2}$ |
| $n_{1}(t),n_{2}(t)$ | Total noise (including all interference components) at receiver $R_{1}$ and $R_{2}$ |
| $g_{1},g_{2}$ | Radiation gains of STC-PM’s for two main spatial modes |
| $g_{i}^{\boldsymbol{r}_{x}\to m\to\boldsymbol{r}_{y}}$ | Radiation gains of STC-PM’s for $i$-th spatial mode for path $\boldsymbol{r}_{x}\to m\to\boldsymbol{r}_{y}$ (Note that $g_{1} = g_{1}^{\boldsymbol{r}_{s}\to m\to\boldsymbol{r}_{1}}, g_{2} = g_{2}^{\boldsymbol{r}_{s}\to m\to\boldsymbol{r}_{2}},$) |
| $g(\boldsymbol{r}_{x},\boldsymbol{r}_{y})$ | Free-space channel gain directly from point $\boldsymbol{r}_{x}$ to $\boldsymbol{r}_{y}$ |
| $c_{1}(t),c_{2}(t)$ | Two temporal modes of STC-PM |
| $s(t;\boldsymbol{r}_{s})$ | Transmitted signal from the designated source $\boldsymbol{r}_{s}$ |
| $s(t;\boldsymbol{r}_{sf})$ | Interference signal transmitted from the interfering source $\boldsymbol{r}_{sf}$ |
| $\tau_{(a\to b)}$ | Propagation delay from point $a$to $b$ |
| $\tau_{m\to\boldsymbol{r}_{u}\to\boldsymbol{r}_{2}}$ | Total round-trip delay of path $m\to\boldsymbol{r}_{u}\to\boldsymbol{r}_{2}$ (Note that $\tau_{m\to\boldsymbol{r}_{u}\to\boldsymbol{r}_{2}} = \tau_{m\to\boldsymbol{r}_{u}} + \tau_{\boldsymbol{r}_{u}\to\boldsymbol{r}_{2}}$) |
| $\alpha_{u}$ | Scattering reflectivity of the target |
| $\boldsymbol{r}_{s}$ | Position of designated signal source |
| $\boldsymbol{r}_{sf}$ | Position of interfering signal source |
| $\boldsymbol{r}_{1},\boldsymbol{r}_{2}$ | Positions of receiver$R_{1}$ and receiver $R_{2}$ |
| $\boldsymbol{r}_{u}$ | Position of the target |
| $N_{r1}(t),N_{r2}(t)$ | Gaussian receiver noise at receivers $R_{1}$ and $R_{2}$ |
| $R_{\tilde{y}_{1},y_{2}}\left( \tau\right)$ | Cross-correlation function between received signals |
| $R_{c_{2}s}(\tau)$ | Cross-correlation between the code $c_{2}(t)$ and the transmitted signal $s(t)$ |

Without loss of generality, in the presence of the interference source $\boldsymbol{r}_{sf}$, we can write out the full expressions of $y_{1}$and $y_{2}$ according to Equation (S27):

| $y_{1}\left( t \right)=g_{1}\text{ }c_{1}\left( t-\tau_{m\to\boldsymbol{r}_{1}} \right)\text{ }s\left( t-\tau_{m\to\boldsymbol{r}_{1}};\boldsymbol{r}_{s} \right)+g_{2}^{\boldsymbol{r}_{s}\to m\to\boldsymbol{r}_{1}}\text{ }c_{2}(t-\tau_{m\to\boldsymbol{r}_{1}})\text{ }s(t-\tau_{m\to\boldsymbol{r}_{1}};\boldsymbol{r}_{s})$  $+g_{1}^{\boldsymbol{r}_{sf}\to m\to\boldsymbol{r}_{1}}c_{1}\left( t-\tau_{m\to\boldsymbol{r}_{1}} \right)\text{ }s\left( t-\tau_{m\to\boldsymbol{r}_{1}};\boldsymbol{r}_{sf} \right)+g_{2}^{\boldsymbol{r}_{sf}\to m\to\boldsymbol{r}_{1}}\text{ }c_{2}(t-\tau_{m\to\boldsymbol{r}_{1}})\text{ }s(t-\tau_{m\to\boldsymbol{r}_{1}};\boldsymbol{r}_{sf})$  $+N_{r1}\left( t \right),$ | (S30) |
| --- | --- |

Therefore, we obtain

| $n_{1}\left( t \right)=g_{2}^{\boldsymbol{r}_{s}\to m\to\boldsymbol{r}_{1}}\text{ }c_{2}(t-\tau_{m\to\boldsymbol{r}_{1}})\text{ }s(t-\tau_{m\to\boldsymbol{r}_{1}};\boldsymbol{r}_{s})$ $+g_{1}^{\boldsymbol{r}_{sf}\to m\to\boldsymbol{r}_{1}}c_{1}\left( t-\tau_{m\to\boldsymbol{r}_{1}} \right)\text{ }s\left( t-\tau_{m\to\boldsymbol{r}_{1}};\boldsymbol{r}_{sf} \right)+g_{2}^{\boldsymbol{r}_{s}\to m\to\boldsymbol{r}_{1}}\text{ }c_{2}(t-\tau_{m\to\boldsymbol{r}_{1}})\text{ }s(t-\tau_{m\to\boldsymbol{r}_{1}};\boldsymbol{r}_{sf})$ $+N_{r1}\left( t \right),$ | (S31) |
| --- | --- |

and,

| $y_{2}\left( t \right)=\underset{Target-scattered STC-\mathrm{PM}-modulated \boldsymbol{r}_{s} signal (Mode 2)}{\underbrace{\alpha_{u}g\left( \boldsymbol{r}_{u},\boldsymbol{r}_{2} \right)g_{2}c_{2}(t-\tau_{m\to\boldsymbol{r}_{u}\to\boldsymbol{r}_{2}})\text{ }s(t-\tau_{m\to\boldsymbol{r}_{u}\to\boldsymbol{r}_{2}};\boldsymbol{r}_{s})}}$  $+\underset{Target-scattered STC-PM-modulated \boldsymbol{r}_{s}\mathrm{signal}\left( Mode1 \right)}{\underbrace{{\alpha_{u}g\left( \boldsymbol{r}_{u},\boldsymbol{r}_{2} \right)g}_{1}^{\boldsymbol{r}_{s}\to m\to\boldsymbol{r}_{u}}\text{ }c_{1}(t-\tau_{m\to\boldsymbol{r}_{u}\to\boldsymbol{r}_{2}})\text{ }s(t-\tau_{m\to\boldsymbol{r}_{u}\to\boldsymbol{r}_{2}};r_{s})}}$  $+ \underset{Target-scattered STC-\mathrm{PM}-modulated \boldsymbol{r}_{sf} signal (Mode 2)}{\underbrace{{\alpha_{u}g\left( \boldsymbol{r}_{u},\boldsymbol{r}_{2} \right)g}_{2}^{\boldsymbol{r}_{sf}\to m\to\boldsymbol{r}_{u}}\text{ }c_{2}(t-\tau_{m\to\boldsymbol{r}_{u}\to\boldsymbol{r}_{2}})\text{ }s(t-\tau_{m\to\boldsymbol{r}_{u}\to\boldsymbol{r}_{2}};\boldsymbol{r}_{sf})}}$  $+\underset{Target-scattered STC-\mathrm{PM}-modulated \boldsymbol{r}_{sf} signal (Mode 1)}{\underbrace{{\alpha_{u}g\left( \boldsymbol{r}_{u},\boldsymbol{r}_{2} \right)g}_{1}^{\boldsymbol{r}_{sf}\to m\to\boldsymbol{r}_{u}}\text{ }c_{1}(t-\tau_{m\to\boldsymbol{r}_{u}\to\boldsymbol{r}_{2}})\text{ }s(t-\tau_{m\to\boldsymbol{r}_{u}\to\boldsymbol{r}_{2}};\boldsymbol{r}_{sf})}}$  $+\underset{\mathrm{Direct} \boldsymbol{r}_{s}\mathrm{signa}l}{\underbrace{g\left( \boldsymbol{r}_{s},\boldsymbol{r}_{2} \right)s\left( t - \left( \tau_{\boldsymbol{r}_{s}\to\boldsymbol{r}_{2}}-\tau_{\boldsymbol{r}_{s}\to m} \right);\boldsymbol{r}_{s} \right)}}+\underset{Target-scattered \mathrm{direct} \boldsymbol{r}_{s}\mathrm{signal}}{\underbrace{g\left( \boldsymbol{r}_{s},\boldsymbol{r}_{u} \right)g\left( \boldsymbol{r}_{u},\boldsymbol{r}_{2} \right)\alpha_{u}s\left( t - (\tau_{\boldsymbol{r}_{s}\to u\to\boldsymbol{r}_{2}}-\tau_{\boldsymbol{r}_{s}\to m});\boldsymbol{r}_{s} \right)}}$  $+\underset{\mathrm{Direct} \boldsymbol{r}_{sf}\mathrm{signa}l}{\underbrace{g\left( \boldsymbol{r}_{sf},\boldsymbol{r}_{2} \right)s\left( t - \left( \tau_{\boldsymbol{r}_{sf}\to\boldsymbol{r}_{2}}-\tau_{\boldsymbol{r}_{sf}\to m} \right);\boldsymbol{r}_{sf} \right)}}$  $+\underset{Target-scattered \mathrm{direct} \boldsymbol{r}_{sf}\mathrm{signal}}{\underbrace{g\left( \boldsymbol{r}_{sf},\boldsymbol{r}_{u} \right)g\left( \boldsymbol{r}_{u},\boldsymbol{r}_{2} \right)\alpha_{u}s\left( t - (\tau_{\boldsymbol{r}_{sf}\to u\to\boldsymbol{r}_{2}}-\tau_{\boldsymbol{r}_{sf}\to m});\boldsymbol{r}_{sf} \right)}}+N_{r2}\left( t \right).$ | (S32) |
| --- | --- |

Accordingly, we can write:

| $n_{2}\left( t \right)={\alpha_{u}g\left( \boldsymbol{r}_{u},\boldsymbol{r}_{2} \right)g}_{1}^{\boldsymbol{r}_{s}\to m\to\boldsymbol{r}_{u}}\text{ }c_{1}\left( t-\tau_{m\to\boldsymbol{r}_{u}\to\boldsymbol{r}_{2}} \right)\text{ }s\left( t-\tau_{m\to\boldsymbol{r}_{u}\to\boldsymbol{r}_{2}};\boldsymbol{r}_{s} \right)$ $+{\alpha_{u}g\left( \boldsymbol{r}_{u},\boldsymbol{r}_{2} \right)g}_{1}^{\boldsymbol{r}_{sf}\to m\to\boldsymbol{r}_{u}}\text{ }c_{1}\left( t-\tau_{m\to\boldsymbol{r}_{u}\to\boldsymbol{r}_{2}} \right)\text{ }s\left( t-\tau_{m\to\boldsymbol{r}_{u}\to\boldsymbol{r}_{2}};\boldsymbol{r}_{sf} \right)$ $+{\alpha_{u}g\left( \boldsymbol{r}_{u},\boldsymbol{r}_{2} \right)g}_{2}^{\boldsymbol{r}_{sf}\to m\to\boldsymbol{r}_{u}}\text{ }c_{2}\left( t-\tau_{m\to\boldsymbol{r}_{u}\to\boldsymbol{r}_{2}} \right)\text{ }s\left( t-\tau_{m\to\boldsymbol{r}_{u}\to\boldsymbol{r}_{2}};\boldsymbol{r}_{sf} \right)$ $+g\left( \boldsymbol{r}_{s},\boldsymbol{r}_{2} \right)s\left( t - \left( \tau_{\boldsymbol{r}_{s}\to\boldsymbol{r}_{2}}-\tau_{\boldsymbol{r}_{s}-m} \right);\boldsymbol{r}_{s} \right)$ $+g\left( \boldsymbol{r}_{s},\boldsymbol{r}_{u} \right)g\left( \boldsymbol{r}_{u},\boldsymbol{r}_{2} \right)\alpha_{u}s\left( t-\left( \tau_{\boldsymbol{r}_{s}\to\boldsymbol{r}_{u}\to\boldsymbol{r}_{2}}-\tau_{\boldsymbol{r}_{s}\to m} \right);\boldsymbol{r}_{s} \right)$ $+g\left( \boldsymbol{r}_{sf},\boldsymbol{r}_{2} \right)s\left( t - \left( \tau_{\boldsymbol{r}_{sf}\to\boldsymbol{r}_{2}}-\tau_{\boldsymbol{r}_{sf}\to m} \right);\boldsymbol{r}_{sf} \right)$ $+g\left( \boldsymbol{r}_{sf},\boldsymbol{r}_{u} \right)g\left( \boldsymbol{r}_{u},\boldsymbol{r}_{2} \right)\alpha_{u}s\left( t-\left( \tau_{\boldsymbol{r}_{sf}\to\boldsymbol{r}_{u}\to\boldsymbol{r}_{2}}-\tau_{\boldsymbol{r}_{sf}\to m} \right);\boldsymbol{r}_{sf} \right)+N_{r2}\left( t \right).$ | (S33) |
| --- | --- |

Beginning with Equation (4) in the main text, the signal components given in Equations (S30) and (S32) are substituted into the numerator of Equation (4), yielding

| $\mathrm{Numerator}of R_{\tilde{y}_{1},y_{2}}\left( \tau\right)$ $= \int\left[ y_{1}\left( t \right)c_{1}^{*}\left( t-\tau_{m\to\boldsymbol{r}_{1}} \right)c_{2}\left( t-\tau_{m\to\boldsymbol{r}_{1}} \right) \right]y_{2}^{*}\left( t +\tau\right)dt$ $\approx\int\left[ g_{1}\text{ }c_{1}\left( t \right)\text{ }s\left( t;\boldsymbol{r}_{s} \right)+n_{1}\left( t \right) \right]c_{1}^{*}\left( t \right)c_{2}\left( t \right)$ $\left[ \alpha_{u}g\left( \boldsymbol{r}_{u},\boldsymbol{r}_{2} \right)\text{ }g_{2}\text{ }c_{2}\left( t+\tau-\tau_{m\to\boldsymbol{r}_{u}\to\boldsymbol{r}_{2}} \right)\text{ }s\left( t+\tau-\tau_{m\to\boldsymbol{r}_{u}\to\boldsymbol{r}_{2}};\boldsymbol{r}_{s} \right)+n_{2}\left( t+\tau\right) \right]^{*}dt$ $=\int\left[ g_{1}\text{ }\left\vert c_{1}\left( t \right) \right\vert^{2}\text{ }s\left( t;\boldsymbol{r}_{\boldsymbol{s}} \right)c_{2}\left( t \right)+n_{1}\left( t \right)c_{1}^{*}\left( t \right)c_{2}\left( t \right) \right]$  $\left[ \alpha_{u}g\left( \boldsymbol{r}_{u},\boldsymbol{r}_{2} \right)\text{ }g_{2}\text{ }c_{2}\left( t+\tau-\tau_{m\to\boldsymbol{r}_{u}\to\boldsymbol{r}_{2}} \right)\text{ }s\left( t+\tau-\tau_{m\to\boldsymbol{r}_{u}\to\boldsymbol{r}_{2}};\boldsymbol{r}_{s} \right)+n_{2}\left( t+\tau\right) \right]^{*}dt$ $+\int g_{1}s\left( t;\boldsymbol{r}_{\boldsymbol{s}} \right)c_{2}\left( t \right)n_{2}^{*}\left( t+\tau\right)dt$ $+ \alpha_{u}^{*}g_{2}^{*}g^{*}\left( \boldsymbol{r}_{u},\boldsymbol{r}_{2} \right)\int n_{1}\left( t \right)c_{1}^{*}\left( t \right)c_{2}\left( t \right)c_{2}^{*}\left( t+\tau-\tau_{m\to\boldsymbol{r}_{u}\to\boldsymbol{r}_{2}} \right)\text{ }s^{*}\left( t+\tau-\tau_{m\to\boldsymbol{r}_{u}\to\boldsymbol{r}_{2}};\boldsymbol{r}_{s} \right)dt$ $+\int n_{1}\left( t \right)c_{1}^{*}\left( t \right)c_{2}\left( t \right)n_{2}^{*}\left( t+\tau\right)dt.$ | (S34) |
| --- | --- |

In Equation (S34), the term $t-\tau_{m\to\boldsymbol{r}_{1}}$ is neglected because its magnitude is small compared with the signal duration. By invoking the orthogonality condition, the direct-path and same-frequency interference terms are analytically removed, leaving only the components associated with the designated source that are modulated by the metasurface. For simplicity, we denote $s(t;r_{s})$ as $s(t)$. In the derivation, we use the relation $\int\left| s\left( t \right) \right|^{2}dt\gg\left| \int c_{2}\left( t \right)n_{2}^{*}\left( t \right)s\left( t \right)dt \right|$,$\left| \int c_{1}^{*}\left( t \right)n_{1}\left( t \right)s^{*}\left( t \right)dt \right|, \left| \int c_{1}^{*}\left( t \right)c_{2}\left( t \right)n_{1}(t)n_{2}^{*}\left( t \right)dt \right|$. Thus,

| $Numerator of R_{\tilde{y}_{1},y_{2}}\left( \tau\right)$ $\approx\alpha_{u}^{*}g_{1}g_{2}^{*}\text{ }g^{*}\left( \boldsymbol{r}_{u},\boldsymbol{r}_{2} \right)\int\left[ s\left( t;\boldsymbol{r}_{s} \right)c_{2}\left( t \right)c_{2}^{*}\left( t+\tau-\tau_{m\to\boldsymbol{r}_{u}\to\boldsymbol{r}_{2}} \right)s^{*}\left( t+\tau-\tau_{m\to\boldsymbol{r}_{u}\to\boldsymbol{r}_{2}};\boldsymbol{r}_{s} \right) \right]dt$ $= \alpha_{u}^{*}g_{1}g_{2}^{*}\text{ }R_{c_{2}s,c_{2}s}\left( \tau-\tau_{m\to\boldsymbol{r}_{u}\to\boldsymbol{r}_{2}} \right)\int\left\vert s\left( t;\boldsymbol{r}_{s} \right) \right\vert^{2}dt,$ | (S35) |
| --- | --- |

where

| \| $R_{c_{2}s,c_{2}s}\left( \tau-\tau_{m\to\boldsymbol{r}_{u}\to\boldsymbol{r}_{2}} \right) = \frac{\int\left[ s\left( t;r_{s} \right)c_{2}\left( t \right)c_{2}^{*}\left( t+\tau-\tau_{m\to\boldsymbol{r}_{u}\to\boldsymbol{r}_{2}} \right)s^{*}\left( t+\tau-\tau_{m\to\boldsymbol{r}_{u}\to\boldsymbol{r}_{2}};\boldsymbol{r}_{s} \right) \right]dt}{\int\left\vert s\left( t;r_{s} \right) \right\vert^{2}dt}.$ \| (S36) \| \| --- \| --- \| | (S36) |
| --- | --- | --- | --- |

In a noise-free environment, the normalization factor is $\int\left| y_{1}\left( t \right)c_{1}^{*}\left( t-\tau_{m\to\boldsymbol{r}_{1}} \right)c_{2}\left( t-\tau_{m\to\boldsymbol{r}_{1}} \right) \right|^{2}dt$, which yields

| $Denominator of R_{\tilde{y}_{1},y_{2}}\left( \tau\right) =g_{1}^{2} \int\left\vert s\left( t;\boldsymbol{r}_{s} \right) \right\vert^{2}dt.$ | (S37) |
| --- | --- |

Therefore,

| $R_{\tilde{y}_{1},y_{2}}\left( \tau\right) =\frac{\alpha_{u}^{*}g_{1}g_{2}^{*}\text{ }R_{c_{2}s}\left( \tau-\tau_{m\to\boldsymbol{r}_{u}\to\boldsymbol{r}_{2}} \right)\int\left\vert s\left( t;\boldsymbol{r}_{s} \right) \right\vert^{2}dt}{g_{1}^{2} \int\left\vert s\left( t;\boldsymbol{r}_{s} \right) \right\vert^{2}dt}= \frac{g_{2}^{*}}{g_{1}} \alpha_{u}^{*} R_{c_{2}s}\left( \tau-\tau_{m\to\boldsymbol{r}_{u}\to\boldsymbol{r}_{2}} \right).$ | (S38) |
| --- | --- |

We now demonstrate that $\int\left| s\left( t \right) \right|^{2}dt\gg\left| \int c_{2}\left( t \right)n_{2}^{*}\left( t \right)s\left( t \right)dt \right|$,$\left| \int c_{1}^{*}\left( t \right)n_{1}\left( t \right)s^{*}\left( t \right)dt \right|, \left| \int c_{1}^{*}\left( t \right)c_{2}\left( t \right)n_{1}(t)n_{2}^{*}\left( t \right)dt \right|$. In what follows, we prove only the first inequality, as the remaining two can be established in an analogous manner.

| $\int c_{2}\left( t \right)n_{2}^{*}\left( t \right)s\left( t \right)dt$ $=\int c_{2}\left( t \right)\left[ {\alpha_{u}g\left( \boldsymbol{r}_{u},\boldsymbol{r}_{2} \right)g}_{1}^{\boldsymbol{r}_{s}\to m\to\boldsymbol{r}_{u}}\text{ }c_{1}\left( t-\tau_{m\to\boldsymbol{r}_{u}\to\boldsymbol{r}_{2}} \right)\text{ }s\left( t-\tau_{m\to\boldsymbol{r}_{u}\to\boldsymbol{r}_{2}};\boldsymbol{r}_{s} \right) \right.$ $+{\alpha_{u}g\left( \boldsymbol{r}_{u},\boldsymbol{r}_{2} \right)g}_{1}^{\boldsymbol{r}_{sf}\to m\to\boldsymbol{r}_{u}}\text{ }c_{1}\left( t-\tau_{m\to r_{u}\to\boldsymbol{r}_{2}} \right)\text{ }s\left( t-\tau_{m\to\boldsymbol{r}_{u}\to\boldsymbol{r}_{2}};\boldsymbol{r}_{sf} \right)$ $+{\alpha_{u}g\left( \boldsymbol{r}_{u},\boldsymbol{r}_{2} \right)g}_{2}^{\boldsymbol{r}_{sf}\to m\to\boldsymbol{r}_{u}}\text{ }c_{2}\left( t-\tau_{m\to\boldsymbol{r}_{u}\to\boldsymbol{r}_{2}} \right)\text{ }s\left( t-\tau_{m\to\boldsymbol{r}_{u}\to\boldsymbol{r}_{2}};\boldsymbol{r}_{sf} \right)$ $+g\left( \boldsymbol{r}_{s},\boldsymbol{r}_{2} \right)s\left( t - \left( \tau_{\boldsymbol{r}_{s}\to\boldsymbol{r}_{2}}-\tau_{\boldsymbol{r}_{s}\to m} \right);\boldsymbol{r}_{s} \right)$ $+g\left( \boldsymbol{r}_{s},\boldsymbol{r}_{u} \right)g\left( \boldsymbol{r}_{u},\boldsymbol{r}_{2} \right)\alpha_{u}s\left( t-\left( \tau_{\boldsymbol{r}_{s}\to\boldsymbol{r}_{u}\to\boldsymbol{r}_{2}}-\tau_{\boldsymbol{r}_{s}\to m} \right);\boldsymbol{r}_{s} \right)$ $+g\left( \boldsymbol{r}_{sf},\boldsymbol{r}_{2} \right)s\left( t - \left( \tau_{\boldsymbol{r}_{sf}\to\boldsymbol{r}_{2}}-\tau_{\boldsymbol{r}_{sf}\to m} \right);\boldsymbol{r}_{sf} \right)$ $\left. +g\left( \boldsymbol{r}_{sf},\boldsymbol{r}_{u} \right)g\left( \boldsymbol{r}_{u},\boldsymbol{r}_{2} \right)\alpha_{u}s\left( t-\left( \tau_{\boldsymbol{r}_{sf}\to\boldsymbol{r}_{u}\to\boldsymbol{r}_{2}}-\tau_{\boldsymbol{r}_{sf}\to m} \right);\boldsymbol{r}_{sf} \right)+N_{r2}\left( t \right) \right]^{*}s\left( t;r_{s} \right) dt$ $\ll\int\left\vert s(t;r_{s}) \right\vert^{2}dt.$ | (S39) |
| --- | --- |

In the above expression, because the factors within each term are mutually orthogonal, each term contributes negligibly (i.e., is close to zero), thus $\ll\int\left| s\left( t \right) \right|^{2}dt$.

**Supplementary Note 5. Analysis of MEPR Detection Performance Under Varying Conditions**

To assess the performance of the proposed MEPR approach, we analyzed its response to point-like targets under different incidence angles, object distances $R$, and aperture ratios $D_{\text{ratio}}$. Here, $D_{\text{ratio}}$ denotes the scaling factor of the aperture dimensions in both x and y directions relative to the original 32 × 24 configuration. The normalized imaging results are presented in Supplementary Fig. 3.

**(1) Angular robustness**

As shown in Supplementary Fig. 3a, the normalized imaging results of point-like objects are presented under different incident angles. Each subfigure corresponds to a specific illumination angle. As the incident angle varies, the reconstructed point images shift accordingly within the field of view, while remaining compact and well focused without noticeable distortion or spreading. These results indicate that the MEPR approach preserves imaging fidelity and maintains strong robustness against angular variation.

**(2) Effect of distance and aperture size**

Supplementary Figs. 3b and 3c show that the imaging resolution gradually degrades as the target distance increases or the aperture size decreases (i.e., with smaller $D_{\mathrm{ratio}}$). As the object moves farther away from the metasurface, the reconstructed spot becomes broader and the 3 dB bandwidth increases accordingly. Likewise, reducing the array dimensions relative to the baseline $32\times24$ configuration weakens the focusing capability and leads to a wider main lobe and lower spatial resolution. These results confirm that a larger effective aperture and a shorter propagation distance both contribute to improved detection accuracy and sharper focusing performance.

###
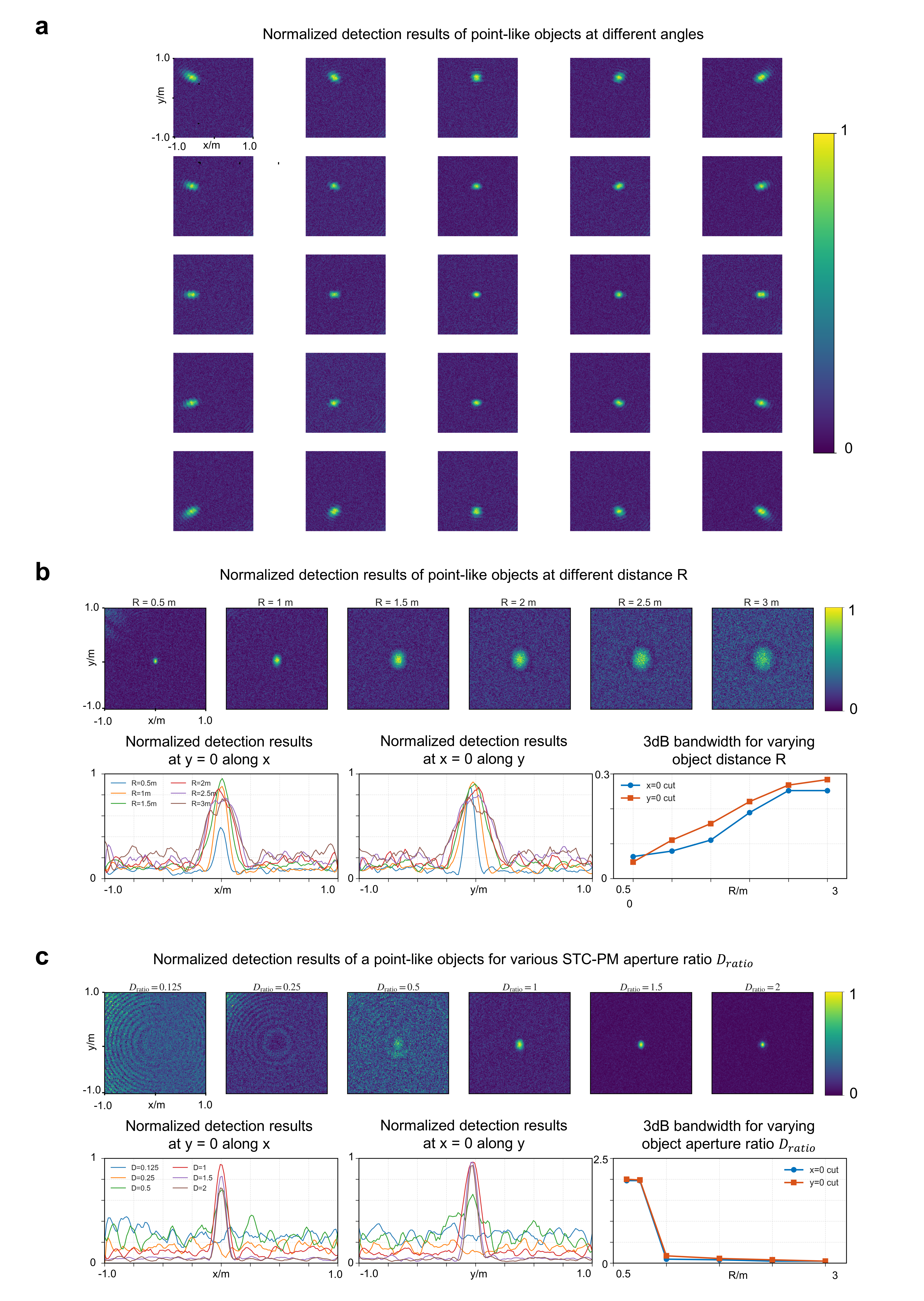
 Supplementary Figure 3 | Normalized imaging results of point-like targets under different conditions. a, Normalized detection maps of point-like targets at different observation angles. Each subpanel presents the reconstructed normalized intensity distribution for a specific angle, demonstrating the angular robustness of the MEPR method. b, Normalized imaging results for point-like targets at different distances $\boldsymbol{R}$. The top row shows reconstructed intensity maps for $\boldsymbol{R}$ = 0.5 m, 1 m, 1.5 m, 2 m, 2.5 m and 3 m. The bottom row presents the corresponding normalized intensity profiles along x at y = 0 (left) and along y at x = 0 (middle), together with the variation of the 3 dB bandwidth as a function of object distance R (right). c, Normalized imaging results for various STC-PM aperture ratios $\boldsymbol{D}_{\text{ratio}}$, defined relative to the baseline 32 × 24 configuration. The top row displays reconstructed intensity distributions for $\boldsymbol{D}_{\text{ratio}}$ $\boldsymbol{=}$ 0.125, 0.25, 0.5, 1,1.5 and 2. The bottom row shows the corresponding normalized intensity profiles along x at y = 0 (left) and along y at x = 0 (middle), together with the 3 dB bandwidth as a function of aperture ratio $\boldsymbol{D}_{\text{ratio}}$ (right).

**Supplementary Note 6. UAV Platform Used in Experiments**

An open-source F290 quadrotor platform was utilized in the experiments (Supplementary Fig. 4a). The UAV has dimensions of $320 mm (H) \times260 mm (W) \times260 mm (L)$, and its primary function is to fly safely and accurately along predefined trajectories in an indoor environment within the vertical plane (the x–y plane defined in Fig. 2a of the main text). The recorded flight trajectories serve as the ground truth for the MEPR system’s tracking performance evaluation.

The system architecture of the UAV is illustrated in Supplementary Fig. 4b. The platform primarily comprises an onboard computer (*Jetson Orin Nano 4G)*, a flight controller (*Pixhawk6C Mini*), and a 3D LiDAR sensor (*MID-360*). The onboard computer handles LiDAR data fusion, visual mapping, and obstacle avoidance, continuously transmitting the UAV’s real-time position to the host PC. It also issues target position commands $(x,y)$ to the flight controller via the MAVROS protocol. The flight controller executes a proportional–integral–derivative (PID) control algorithm to compute the necessary attitude and thrust adjustments, generating the corresponding motor control signals. These signals are then sent to the electronic speed controllers (ESCs), which translate them through pulse-width modulation (PWM) or DShot protocols into drive voltages for the motors. Four *T-Motor F100 KV1100* brushless motors provide lift and are arranged in alternating clockwise and counterclockwise orientations to maintain torque balance. As the UAV lacks a GPS module, all operations are confined to indoor environments. To ensure safe operation, the onboard computer enforces software-defined flight boundaries; if the UAV deviates from the designated area or detects hardware anomalies, it automatically returns to its starting position.

We developed a trajectory generation algorithm specifically designed to produce alphabet-shaped flight paths in the vertical plane, as illustrated in Supplementary Fig. 4c. Each English letter was first converted into a binary image, from which its skeleton was extracted to define discrete waypoints representing the geometric outline of the letter. Morphological filtering was then applied to enhance the trajectory’s continuity and smoothness. The resulting path points were subsequently normalized and transformed into the UAV’s coordinate frame. These preconfigured trajectories were sequentially uploaded as position commands, enabling the UAV to accurately reproduce letter shapes (e.g., “P,” “K,” “U,” “E,” “R,” and “S”). During flight, the 3D LiDAR sensor continuously recorded the UAV’s real-time motion data, which served as reference traces for performance evaluation. The corresponding letter trajectories are depicted at the bottom of Supplementary Fig. 4c.

To enable synchronized control and data acquisition, a unified desktop control interface was developed using the Vue framework (Supplementary Fig. 4d). The interface integrates all operational modules of the UAV system, allowing real-time command transmission, state monitoring, and data visualization within a single control panel. During experiments, the interface facilitated seamless coordination between the UAV, onboard computer, and host PC, ensuring stable communication and precise execution of preconfigured flight paths while recording all relevant experimental data for subsequent analysis.





### Supplementary Figure 4 | Experimental UAV platform and system architecture. a, Experimental UAV platform (open-source F290 frame) equipped with a Pixhawk 6C Mini flight controller, a 3D LiDAR (MID-360) for spatial perception, and an onboard Jetson Orin Nano (4 GB) computer. The overall dimensions of the UAV are 320 mm (H) × 260 mm (W) × 260 mm (L). b, System architecture showing the data and control flow among the 3D LiDAR, onboard computer, flight controller, and ESC & motor units. c, Workflow for generating preconfigured letter-shaped flight trajectories. The example shows the letter “P”: starting from a font template, skeleton extraction and morphological filtering are applied to derive smooth, time-normalized trajectories. The resulting trajectories for representative letters (“P”, “K”, “U”, “E”, “R”, “S”) are displayed. d, Vue-based UAV takeoff and control interface enabling real-time mission setup and parameter editing.

**Supplementary Note 7. Greedy Search Algorithm for Rapid UAV Detection and Tracking**

***Wireless Signal Detection and Far-Field Angular Scanning***

As described in Supplementary Note 1, the wireless signal reception and far-field angular scanning are coordinated by a hierarchical timing-control architecture. The personal computer (PC) communicates with the FPGA via Ethernet and with the USRP via optical fiber to configure the spatiotemporal coding sequences and timing schedules. The USRP serves as the hardware-level timing reference and generates microsecond-scale GPIO trigger pulses that synchronize the FPGA’s coding-state transitions and signal acquisition. This design ensures precise temporal alignment and real-time operation throughout the entire spatiotemporal modulation process.

**Supplementary Figure 5 | Barker code–based signal detection and synchronization process.** **a,** Transmitted 13-bit Barker sequence $[1,1,1,1,1,-1,-1,1,1,-1,1,-1,1]$, where each element (“+1” or “−1”) controls the metasurface reflection phase toward receiver $R_{1}$. **b,** Normalized received power $y_{1}(t)$, showing a clear increase when the external signal is present. The red dashed line marks the detected matched point. **c,** Cross-correlation result between the received signal and the reference Barker code, where a sharp correlation peak indicates a successful detection and timing synchronization of the external emission. The Barker code’s low sidelobe and high main-lobe characteristics provide strong robustness against noise and asynchronous signal arrivals. In practice, zero-padding is applied on both sides of the 13-bit sequence to form a 100-bit recurring Barker cycle for consistent frame synchronization.


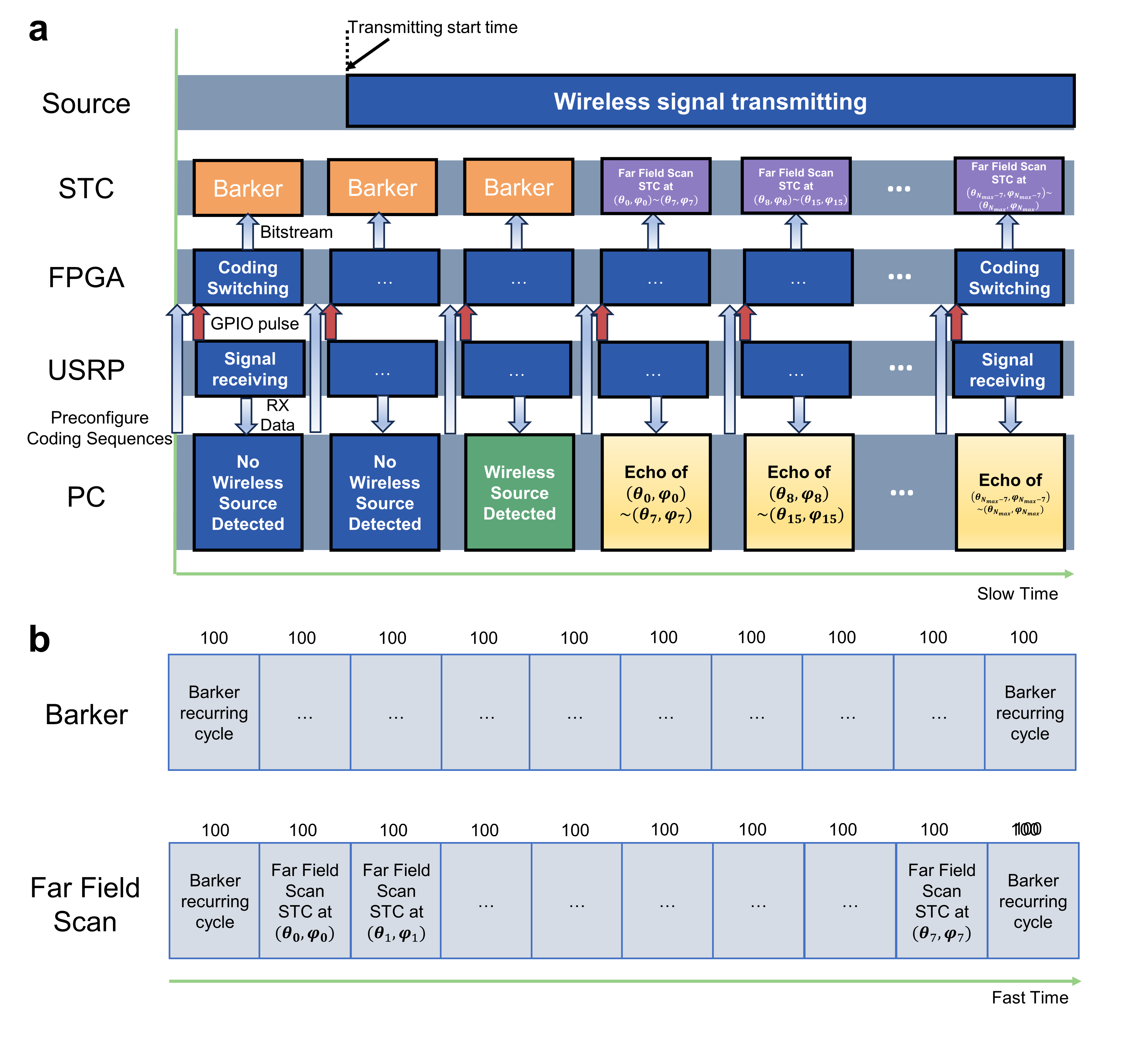


**Supplementary Figure 6 | Wireless signal detection and far-field angular scanning process. a,** System architecture showing the temporal coordination among the PC, USRP, FPGA, and STC-PM during Barker-based signal detection and subsequent far-field angular scanning. **b,** Timing diagram illustrating the STC sequence, including the 13-bit Barker code for source detection and the subsequent far-field scanning cycles used for target localization.

During each STC cycle, the system operates as follows. The PC first preloads a one-bit bitstream of dimension $32 \times24 \times1000$ into the FPGA. The USRP then issues a GPIO trigger to initiate the STC sequence. Under FPGA control, the PM performs 1000 coding transitions at a rate of 2.5 µs per code, while the USRP simultaneously acquires signals from two receiving channels ($R_{1}$ and $R_{2}$) at a 10 MHz sampling rate, yielding 25,000 samples per acquisition window. The tight synchronization between STC modulation and data acquisition maintains phase coherence at the microsecond scale.

Because the emission timing of external wireless sources is typically unknown in MEPR environments, a fast signal-detection mechanism based on a 13-bit Barker sequence $[1,1,1,1,1,-1,-1,1,1,-1,1,-1,1]$is employed to ensure robust synchronization under non-cooperative or burst-signal conditions. In practice, zero-padding is applied on both sides of the 13-bit sequence to form a 100-bit recurring Barker cycle for consistent frame synchronization. As shown in Supplementary Fig. 5a, the metasurface sequentially transmits this Barker-coded excitation, where each code element (“+1” or “−1”) determines whether energy is directed toward receiver $R_{1}$. Before any external signal is received, the metasurface continuously cycles through the Barker sequence to maintain awareness of the surrounding wireless field. When an incoming waveform is present, its normalized power increases sharply (Supplementary Fig. 5b). Through cross-correlation with the reference Barker code (Supplementary Fig. 5c), a strong correlation peak appears precisely at the matched delay point, confirming the presence and timing of a valid source signal. Once this peak is detected, the system automatically initiates far-field angular scanning to perform sensing tasks. The Barker code is selected for its excellent autocorrelation properties (characterized by a narrow main lobe and low sidelobes) which provide high resilience against noise and asynchronous signal arrivals.

After signal detection, the system transitions to far-field angular scanning. Following the STC design described in the main text, the PM operates in two spatial modes. Mode 1 establishes a stable link between the metasurface and receiver $R_{1}$, producing the modulated reference signal. Mode 2 forms a scanning beam directed toward far-field angular coordinates $(\theta, \varphi$) for target probing. Each angular position is scanned using a spatiotemporal coding sequence of length $T_{L}=100T_{c}$, which provides an effective balance between clutter suppression and time consumption. In implementation, the FPGA sequentially loads predefined code sequences to steer the metasurface beams across discrete angular steps, while the USRP receiver performs the signal processing defined by Equation (4) in the main text on the acquired echoes to obtain normalized echo intensities, enabling reliable detection even in the presence of interference.

Supplementary Figs. 6a and 6b illustrate the timing coordination among the system modules. Supplementary Fig. 6a presents the hierarchical synchronization between the PC, USRP, FPGA, and metasurface, showing the progression from Barker-based source detection to angular scanning. Supplementary Fig. 6b details the timing cycles of Barker code recurrence and far-field scanning in both slow and fast-time domains, demonstrating the precise temporal alignment achieved by the system. This framework enables an interference-resilient sensing architecture capable of rapid, autonomous angular-domain detection and tracking in multipath and asynchronous environments.





**Supplementary Figure 7 | Greedy search–based UAV detection and tracking algorithm. a,** System architecture and flowchart of the greedy search algorithm implemented on the PC. The metasurface, controlled by the MCU and FPGA through the USRP, performs sequential angular-domain scans. The algorithm operates in four stages: *Wireless Source Detection*, *Background Scan*, *Coarse Scan with Background Subtraction*, and *Fine Scan with Tracking*. During initialization, the system constructs a background map by sequentially loading predefined coding patterns to record static reflections. In the coarse-scan stage, sparse angular sampling is performed to detect potential target directions by subtracting the background response from the current measurement. Once regions with significant echo enhancement are identified, the fine-scan stage conducts dense angular sampling to obtain a high-resolution local echo map. The detection confidence is evaluated from the maximum normalized response, and when it exceeds the preset threshold, the target position is estimated using a weighted centroid approach. The centroid positions are iteratively updated in subsequent frames through a greedy search–based prediction loop. When the detection confidence remains below threshold for several consecutive frames, the system automatically re-enters the coarse-scanning phase to reacquire the target. **b,** Experimental demonstration of the scanning and tracking process. The four panels show normalized echo intensity maps obtained at different stages: *Background Scan*, *Coarse Scan after Background Subtraction*, and *Fine Scan*. The progressive refinement illustrates the improvement in localization accuracy and signal-to-noise ratio, leading to continuous and reliable tracking of the UAV trajectory.

***Greedy Search Algorithm***

The algorithmic procedure is illustrated in Supplementary Fig. 7.

**(1) Wireless Source Detection and Background Scanning**

At the initialization stage, the system configures the STC sequence in Barker Recycle mode to detect the presence of a wireless source. If the correlation response $R_{d}=max[y_{1}(t)*\text{Barker}(t-\tau)]$exceeds a detection threshold, the system confirms the existence of a dynamic source and proceeds to scanning; otherwise, it remains in the wireless source detection mode.

During background scanning, the STC-PM sequentially loads predefined coding sequences for far-field position $(\theta_{i},\varphi_{i})$ to cover the desired angular domain. The receiver records the echo responses $y_{1}(t)$and $y_{2}(t)$in the absence of a target to form a background reflection map: $R_{bg}(\theta_{i},\varphi_{i})$. This map captures reflections from static structures and serves as a reference for subsequent subtraction, improving SNR and suppressing false alarms.

**(2) Coarse Scanning and Background Subtraction**

Once the wireless source is detected, the system switches to a coarse scanning phase to rapidly localize the target region. Sparse angular-domain sampling is performed, and for each beam direction the coarse-scan response $R_{c}(\theta_{i},\varphi_{i})$is computed and background-subtracted:
 $R(\theta_{i},\varphi_{i})=R_{c}(\theta_{i},\varphi_{i})-R_{bg}(\theta_{i},\varphi_{i})$ (S40)

Candidate regions with strong residual responses (confidence > Threshold₂) are then extracted for further refinement.

The complete procedure is summarized in Algorithm 1 (Coarse Position Track).


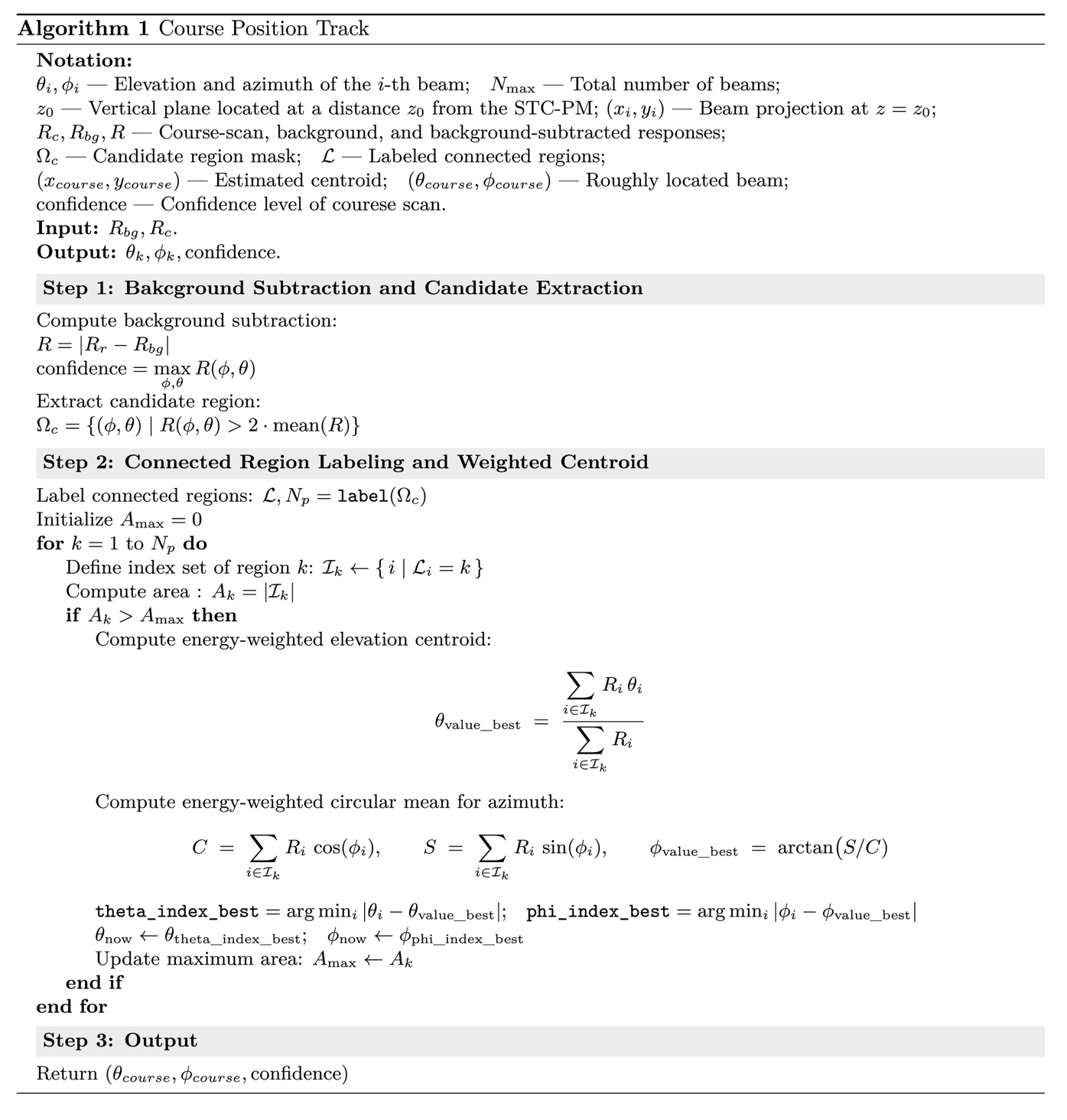


**(3) Fine Scanning and Position Estimation**

Within the candidate region, the system performs a fine scanning process using dense angular sampling to obtain high-resolution spatial localization. The fine-scan responses are calculated and the target position is refined based on energy-weighted centroid estimation.

The detailed fine-scan process, including geometric projection, candidate beam selection, and angular refinement, is summarized in Algorithm 2 (Fine Position Track).

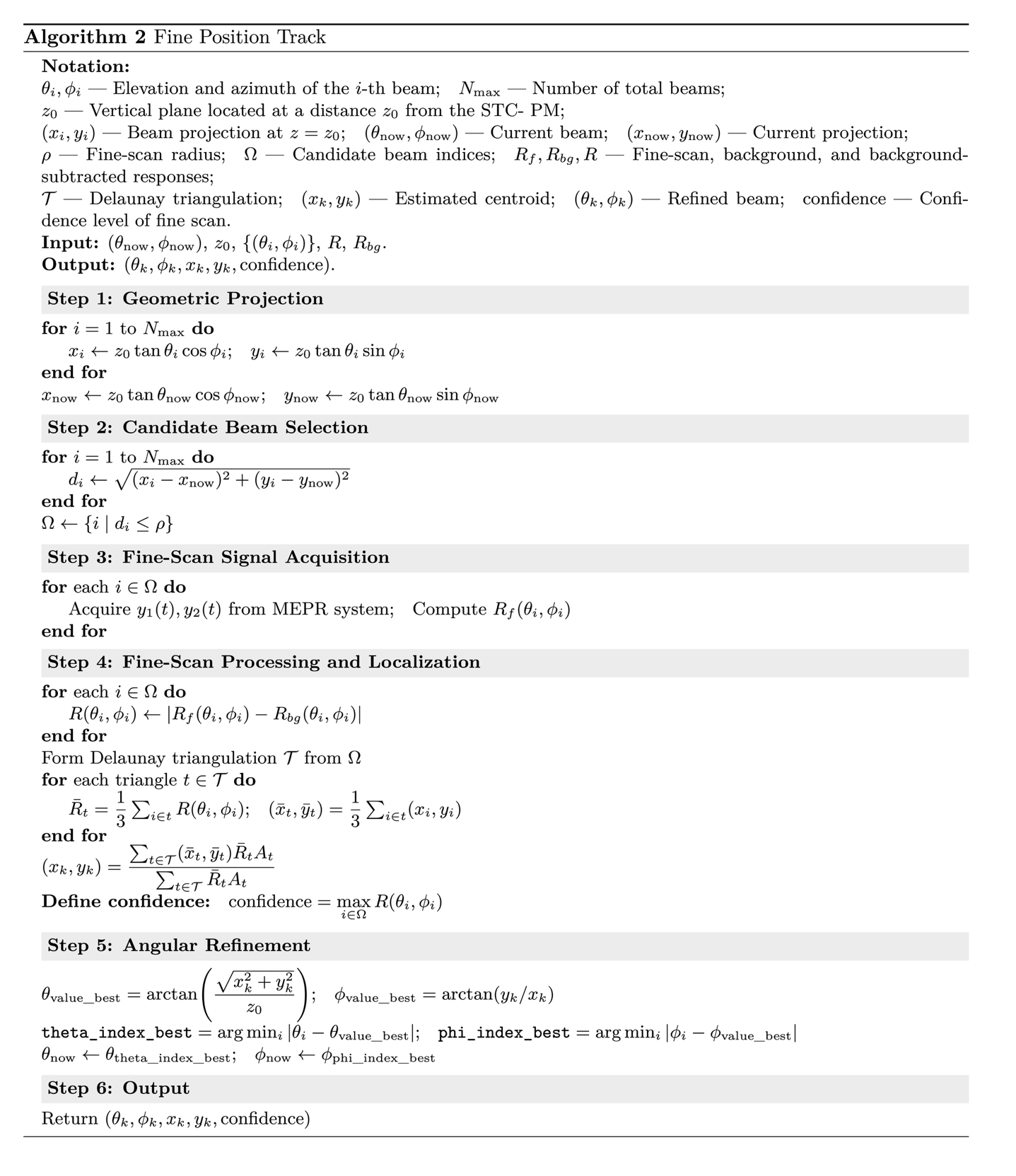


**(4) Dynamic Tracking and Adaptive Switching**

After initial localization, the system transitions into a greedy search–based dynamic tracking mode. The algorithm predicts the target’s probable direction in the next frame from the temporal evolution of the previously estimated centroids. A local scanning window centered on the predicted direction is dynamically adjusted, enabling adaptive fine scanning with reduced computational cost. When the detection confidence remains above a threshold, the UAV trajectory is continuously reconstructed in real time, as illustrated in the **“**Tracking Result**”** panel of Supplementary Fig. 7a. If the confidence drops below the threshold for several consecutive frames, the system automatically reverts to the coarse scanning phase for target reacquisition, ensuring robust operation across detection and tracking stages.

**Supplementary Note 8. Link-budget Analysis for MEPR**

This supplementary note presents a simplified link-budget analysis for the proposed metasurface-enabled passive imaging method to evaluate the output SINR after code-aligned correlation processing and thereby estimate the achievable detection range; for simplicity, the following derivation considers only direct-path interference and thermal noise, while co-channel interference is not included.

We consider the discrete-time received signals in the reference and surveillance channels as

| $R_{1}[n]=h_{1}c_{1}[n]s[n]+N_{r1}[n],$ | (S41) |
| --- | --- |

And

| $R_{2}[n]=h_{2}c_{2}[n]s[n]+h_{d}s[n]+N_{r2}[n],$ | (S42) |
| --- | --- |

where $n=1,2,\ldots,N$, with $N=BT$ denoting the number of effective samples collected over bandwidth $B$ and observation time $T$ of the designated ambient signal, respectively. Here, $s[n]$ is the ambient signal, and $c_{1}[n]$ and $c_{2}[n]$ are the temporal coding sequences associated with the reference and probing channels, respectively. $h_{1}$ denotes the effective complex channel coefficient of the STC-PM-modulated reference-path signal, namely, the signal component received at the reference receiver through temporal mode 1. $h_{2}$ denotes the effective complex channel coefficient of the desired target-echo component received at the surveillance receiver through temporal mode 2. In addition, $h_{d}$ represents the effective complex response of the undesired component, including the direct-path signal and the direct-wave-induced scattered contribution. The additive terms $N_{r1}[n]$ and $N_{r2}[n]$ denote additive white Gaussian noise at the receivers. To facilitate the subsequent analysis, we define the effective power gains corresponding to the above channel coefficients as

| $G_{1}=\vert h_{1}\vert^{2}, G_{2}=\vert h_{2}\vert^{2}, G_{d}=\vert h_{d}\vert^{2}.$ | (S43) |
| --- | --- |

The proposed imaging method is based on code-aligned cross-correlation. We define the code-aligned cross-correlation term

| $a[n]=R_{1}[n]R_{2}^{*}[n]c_{1}^{*}[n]c_{2}[n],$ | (S44) |
| --- | --- |

and the accumulated detection statistic as

| $A=\sum_{n=1}^{N} a[n].$ | (S45) |
| --- | --- |

Substituting the received signals into the above expression gives

| $A=A_{\mathrm{sig}}+A_{n1}+A_{n2}+A_{\mathrm{nn}}+A_{d}+A_{\mathrm{sn}},$ | (S46) |
| --- | --- |

where the desired contribution is

| $A_{\mathrm{sig}}=h_{1}h_{2}^{*}\sum_{n=1}^{N} \vert s[n]\vert^{2},$ | (S47) |
| --- | --- |

and the undesired terms are given by

| $A_{n1}=h_{2}^{*}\sum_{n=1}^{N} N_{r1}[n]c_{1}^{*}[n]s^{*}[n],$ | (S48) |
| --- | --- |
| $A_{n2}=h_{d}^{*}\sum_{n=1}^{N} N_{r1}[n]c_{1}^{*}[n]c_{2}[n]s^{*}[n],$ | (S49) |
| $A_{\mathrm{nn}}=\sum_{n=1}^{N} N_{r1}[n]N_{r2}^{*}[n]c_{1}^{*}[n]c_{2}[n],$ | (S50) |
| $A_{d}=h_{d}^{*}h_{1}\sum_{n=1}^{N} \left\vert s[n] \right\vert^{2}c_{2}[n],$ | (S51) |

and

| $A_{\mathrm{sn}}=h_{1}\sum_{n=1}^{N} s[n]c_{2}[n]N_{r2}^{*}[n].$ | (S52) |
| --- | --- |

The term $A_{\mathrm{sig}}$ is the useful component that is fully aligned with the code projection, while the remaining terms arise from receiver noise and uncoded direct-path-related interference. The key mechanism is that the desired contribution is coherently accumulated after code matching, whereas the interference term $A_{d}$ is not fully aligned with the temporal code and therefore accumulates much less efficiently.

To evaluate the correlation-domain signal-to-interference-plus-noise ratio, we assume that the designated ambient signal is a zero-mean circularly symmetric complex Gaussian random process,

| $s[n\mathcal{]\sim CN}(0,\sigma_{s}^{2}),$ | (S53) |
| --- | --- |

and define

| $P_{s}\mathbb{=E\{\vert}s[n]\vert^{2}\}.$ | (S54) |
| --- | --- |

Under this assumption,

| $\mathbb{E\{\vert}s[n]\vert^{4}\}=2P_{s}^{2}.$ | (S55) |
| --- | --- |

We further denote the receiver-noise powers by

| $P_{N_{1}}\mathbb{=E\{\vert}N_{r1}[n]\vert^{2}\}, P_{N_{2}}\mathbb{=E\{\vert}N_{r2}[n]\vert^{2}\}.$ | (S56) |
| --- | --- |

The energy of the desired term can be written as

| $\mathbb{E\{\vert}A_{\mathrm{sig}}\vert^{2}\}=\vert h_{1}\vert^{2}\vert h_{2}\vert^{2}\mathbb{E}\left\{ \left\vert\sum_{n=1}^{N} \vert s[n]\vert^{2} \right\vert^{2} \right\}.$ | (S57) |
| --- | --- |

Using the fourth-order moment of the complex Gaussian signal, we obtain

| $\mathbb{E\{\vert}A_{\mathrm{sig}}\vert^{2}\}=\vert h_{1}\vert^{2}\vert h_{2}\vert^{2}\left[ N\mathbb{E\{\vert}s[n]\vert^{4}\}+N(N-1)(\mathbb{E\{\vert}s[n]\vert^{2}\})^{2} \right],$ | (S58) |
| --- | --- |

which yields

| $\mathbb{E\{\vert}A_{\mathrm{sig}}\vert^{2}\}=N(N+1)P_{s}^{2}G_{1}G_{2}.$ | (S59) |
| --- | --- |

This expression shows that the desired response enjoys coherent accumulation over the entire observation window.

The remaining terms contribute to the interference and noise floor. Their energies are

| $\mathbb{E\{\vert}A_{n1}\vert^{2}\}\boldsymbol{=}\left\vert h_{2} \right\vert^{2}\mathbb{E}\left\{ \left\vert\sum_{n=1}^{N} N_{1}\left[ n \right]c_{1}^{*}\left[ n \right]s^{*}\left[ n \right] \right\vert^{2} \right\}$  $= \left\vert h_{2} \right\vert^{2}\mathbb{E}\left( \sum_{n=1}^{N} \left\vert N_{r1}\left[ n \right] \right\vert^{2}\left\vert s\left[ n \right] \right\vert^{2} \right) = NP_{s}P_{N_{r1}}G_{2}$ | (S60) |
| --- | --- |
| $\mathbb{E\{\vert}A_{n2}\vert^{2}\}=NP_{s}P_{N_{r1}}G_{d},$ | (S61) |
| $\mathbb{E\{\vert}A_{\mathrm{nn}}\vert^{2}\}=NP_{N_{r1}}P_{N_{r2}},$ | (S62) |

and

| $\mathbb{E\{\vert}A_{\mathrm{sn}}\vert^{2}\}=NP_{s}P_{N_{r2}}G_{1}.$ | (S63) |
| --- | --- |

The residual direct-path-related interference term is special because its accumulation is partially reduced by the temporal coding sequence. Its energy becomes

| $\mathbb{E\{\vert}A_{d}\vert^{2}\}=\vert h_{d}\vert^{2}\vert h_{1}\vert^{2} \mathbb{E}\left\{ \left\vert\sum_{n=1}^{N} \left\vert s[n] \right\vert^{2}c_{2}[n] \right\vert^{2} \right\}$ $= \vert h_{d}\vert^{2}\vert h_{1}\vert^{2}\left[ N\mathbb{E}\left\{ \left\vert s\left[ n \right] \right\vert^{4} \right\} + 2\sum_{i<j} \mathbb{E}\left\{ \left\vert s\left[ i \right] \right\vert^{2}\left\vert s\left[ j \right] \right\vert^{2}c_{2}\left[ i \right]c_{2}^{*}\left[ j \right] \right\} \right]$ $= \vert h_{d}\vert^{2}\vert h_{1}\vert^{2}\left[ N\cdot2P_{s}^{2} + M\cdot\frac{N}{M}\cdot(\frac{N}{M}-1)P_{s}^{2} \right]$ $= N(\frac{N}{M}+1)P_{s}^{2}G_{d}G_{1}.$ | (S64) |
| --- | --- |

where $M$ is the temporal coding length of the STC-PM.

Combining the useful and undesired terms, the output SINR after code-aligned cross-correlation is given by

| $SINR=\frac{N(N+1)P_{s}^{2}G_{1}G_{2}}{N\left( \frac{N}{M}+1 \right)P_{s}^{2}G_{d}G_{1}+NP_{s}P_{N_{r1}}G_{2}+NP_{s}P_{N_{r1}}G_{d}+NP_{N_{r1}}P_{N_{r2}}+NP_{s}P_{N_{r2}}G_{1}}.$ | (S65) |
| --- | --- |

Equivalently, after normalization by ${NP_{s}G}_{1}$, the above expression can be rewritten as

| $SINR=\frac{(N+1)G_{2}P_{s}}{\left( \frac{N}{M}+1 \right)G_{d}P_{s}+\frac{P_{N_{r1}}}{G_{1}P_{s}}G_{2}P_{s}+\frac{P_{N_{r1}}}{G_{1}P_{s}}G_{d}P_{s}+\frac{P_{N_{r1}}P_{N_{r2}}}{G_{1}P_{s}}+P_{N_{r2}}}.$ | (S66) |
| --- | --- |

From Equation (S66), it can be seen that part of the STC-PM aperture is used to establish a stable wireless link between a selected ambient source and the reference receiver, thereby providing a relatively clean observation of the ambient signal in the reference channel. When the reference-channel signal-to-noise ratio is sufficiently high, namely, $\frac{P_{N_{r1}}}{G_{1}P_{s}}\ll1,$ the three terms $\frac{P_{N_{r1}}}{G_{1}P_{s}}G_{2}P_{s}$, $\frac{P_{N_{r1}}}{G_{1}P_{s}}G_{d}P_{s}$, $\frac{P_{N_{r1}}P_{N_{r2}}}{G_{1}P_{s}}$, become negligible. In this case, the output SINR can be further approximated as

| $SINR\approx\frac{(N+1)G_{2}P_{s}}{\left( \frac{N}{M}+1 \right)G_{d}P_{s}+P_{N_{r2}}}.$ | (S67) |
| --- | --- |

To further interpret Equation (S67), we define an effective output signal-to-noise ratio (SNR) and signal-to-interference ratio (SIR) as

| $SNR=\frac{(N+1)G_{2}P_{s}}{P_{N_{r2}}}, SIR=\frac{(N+1)G_{2}P_{s}}{\left( \frac{N}{M}+1 \right)G_{d}P_{s}}.$ | (S68) |
| --- | --- |

Accordingly, the output SINR may be regarded as being approximately limited by the smaller of these two quantities, i.e.,

| $SINR\sim min\{SNR,SIR\}.$ | (S69) |
| --- | --- |

To further clarify the achievable detection range of the proposed method, we performed new numerical calculations and summarized the results in Supplementary Fig. 8. This figure shows the maximum detectable UAV distance, $R_{PM\leftrightarrow UAV,max}$, of the proposed method under different temporal coding lengths $M$, metasurface sizes $N$, and source-to-metasurface distances $R_{S\leftrightarrow PM}$. Here, $R_{PM\leftrightarrow UAV,max}$ is defined as the maximum detectable distance for which the SINR remains no lower than 10 dB. We considered a representative operating scenario at 5.48 GHz, assuming an ambient-source EIRP of 42 dBm, a receiver gain of 6 dBi, a representative UAV radar cross section of -15 dBsm, an STC-PM insertion loss of 3 dB, an additional system loss of 3 dB, a bandwidth of 20MHz, and a receiver noise figure of 5 dB.

As shown in Supplementary Fig. 8, for a fixed temporal coding length and metasurface aperture size, $R_{PM\leftrightarrow UAV,max}$ decreases monotonically as $R_{S\leftrightarrow PM}$ increases, confirming the reviewer’s concern that the additional propagation segment reduces the effective sensing range. At the same time, Supplementary Fig. 8 also shows that this loss can be substantially compensated by increasing either the temporal coding length or the metasurface aperture. Notably, even with a relatively modest aperture size of $N=2000$, when $M=1000$, $R_{S\leftrightarrow PM}$ and $R_{PM\leftrightarrow UAV,max}$ can exceed 30 m, which is already sufficient for short-to-medium-range low-altitude sensing and surveillance scenarios.

**Supplementary Figure 8 |** **Maximum detectable UAV distance versus source-to-metasurface distance.** Numerically calculated maximum detectable UAV distance, $R_{PM\leftrightarrow UAV,max}$, versus source-to-metasurface distance, $R_{S\leftrightarrow PM}$, for different temporal coding lengths $M$ and metasurface sizes $N$ of the STC-PM. The maximum detectable distance is defined under a 10 dB SINR criterion.
